# A woman with diabetes presenting with pyomyoma and treated with subtotal hysterectomy: a case report

**DOI:** 10.4076/1752-1947-3-7439

**Published:** 2009-09-08

**Authors:** Horace Fletcher, Racquel Gibson, Nadia Williams, Gilian Wharfe, Allison Nicholson, Deanne Soares

**Affiliations:** 1 Department of Obstetrics and Gynaecology, University of the West Indies, Mona, Kingston 7, Jamaica, West Indies; 2 Department of Pathology, University of the West Indies, Mona, Kingston 7, Jamaica, West Indies; 3 Department of Microbiology, University of the West Indies, Mona, Kingston 7, Jamaica, West Indies; 4 Department of Radiology, University of the West Indies, Mona, Kingston 7, Jamaica, West Indies

## Abstract

**Introduction:**

Pyomyoma (suppurative leiomyoma of the uterus) is a rare condition resulting from infarction and infection of a leiomyoma. It is more usual in pregnant women or postmenopausal women who have vascular disease. The condition is usually fatal unless treated with appropriate antibiotics and surgical intervention.

**Case presentation:**

We report a case of a 44-year-old Afro-Caribbean woman with diabetes who presented with recurrent episodes of abdominal pain and fever over a period of five months. Her problem proved to be a diagnostic dilemma mimicking cholecystitis, pyelonephritis and ovarian cancer. Her blood cultures were positive on one occasion for methicillin-resistant *Staphylococcus epidermidis*. An ultrasound scan suggested uterine fibroids but a computed tomography scan suggested an ovarian malignancy because the mass appeared heterogeneous with fluid filled areas. She was treated with several courses of antibiotics and eventually at laparotomy, she was found to have a large pyomyoma which was successfully removed by subtotal hysterectomy with immediate and complete resolution of her symptoms.

**Conclusion:**

The diagnosis of pyomyoma should be considered in perimenopausal women with large fibroids and pyrexia of unknown origin.

## Introduction

Uterine fibroids are very common, with small leiomyomas present in more than 20% of women over the age of 40 years, which usually remain asymptomatic [1]. Thus the recommendation in most women with fibroids in the perimenopausal period is to be conservative. However, large fibroids found in these women may cause infection [2]. Infection (suppurative leiomyoma of the uterus) is more common in these women because of vascular disease which causes infarction in the fibroids [2]. Women with large fibroids and comorbid conditions such as diabetes, who present with pyrexia of unknown origin, should be suspected as having this unusual serious condition until it is proven otherwise. Removal of asymptomatic large fibroids in perimenopausal women may therefore be necessary since these patients are not without risk as was previously thought.

## Case presentation

The patient was a 44-year-old Afro-Caribbean woman who was diabetic and receiving oral metformin treatment. She was referred to the Accident and Emergency department at the University Hospital of the West Indies with a one day history of right upper quadrant pain, diarrhoea, numerous bouts of vomiting and fever. She had a history of uterine fibroids but had normal menstrual cycles, with her last period being one month before presentation. Her last sexual activity was before her menses. There was no abdominal or pelvic surgical intervention before her presentation. The provisional diagnosis was acute cholecystitis. When examined, it was noted that she was febrile and tachycardic. A non-tender abdominal mass was also noted filling the abdomen extending to the right upper quadrant. There was no renal angle tenderness and only mild tenderness in the right upper quadrant.

Her blood sugar was very high at 453.6 mg/100 mL (normal 75-115 mg/100 mL) and serology for HIV was negative. An ultrasound scan revealed a normal gall bladder, kidneys, liver and spleen. A large uterus with heterogeneous echo pattern was noted suggesting multiple uterine fibroids. The mass in the right upper quadrant appeared to be connected to the uterus and was thought most likely to be a subserosal fibroid. The patient was seen by the medical resident on-call and treated for her uncontrolled diabetes, placed on norfloxacin and sent home. While at home she remained unwell and was treated by her general practitioner as an outpatient for three weeks with oral antibiotics for "a right pyelonephritis which would not resolve". She was thus sent back to hospital and on this occasion she was described by the resident on-call as "not very ill-looking" with a pulse rate of 135/minute, BP 129/71, temp 38ºC, respiratory rate of 20/minute. Her chest examination revealed basal crepitations in the right lower zone and her abdominal examination again revealed a large 34 week size mass arising from the pelvis described this time as asymptomatic. Her blood test results revealed blood sugar of 414 mg/100 mL (normal 75-115 mg/100 mL), haemoglobin (Hb) 7.8 g/dL (normal range 11-15 g/dL), white blood cell count (WBC) 22.5 × 10^9^/L (normal range 4-11 × 10^9^/L) with neutrophilia, platelets 315 × 10^9^/L (normal range 150-450 × 10^9^/L) and a mild elevation of both her urea and creatinine. She also had a collagen vascular screen which was normal except for a mild elevation of her rheumatoid factor of 59.9 IU/mL (normal <40). Blood and urine cultures were both sterile and she was admitted for intravenous antibiotic treatment and insulin control of her diabetes. Treatment with ceftriaxone, amoxicillin and clavulanic acid was started and over a six-day period she continued to have temperature spikes with fever up to 39ºC. She eventually became afebrile on day seven and after two additional days of antibiotics, she was sent home with oral antibiotic treatment and her oral antidiabetic medications were restarted. The patient was discharged with good diabetic control and was feeling much better.

**Figure 1:**
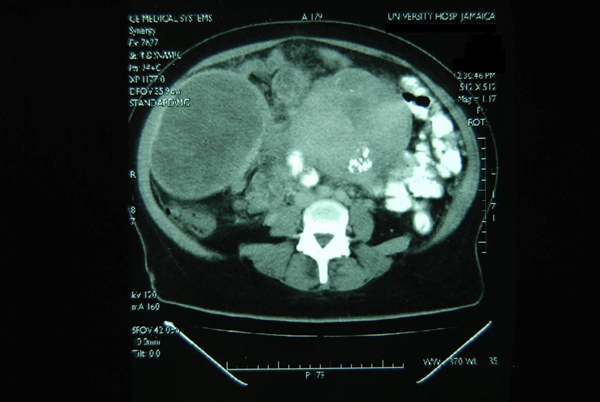
**A computed tomography scan showing a large multicystic mass (15**.5 × 16 × 9 cm) adherent to the abdominal wall.

During her admission, a repeat ultrasound scan revealed a large heterogeneous mass with central cystic areas intimately related to the uterus with a diagnosis of possibly uterine fibroids. A computed tomography (CT) scan was also done with and without intravenous contrast to evaluate the abdominal mass. This showed a large multicystic mass 15.5 × 16 × 9 cm extending up to the right inferior border of the liver, suggestive of an ovarian cancer. The mass was said to be intimately adherent to the anterior abdominal wall (Figure 1). A Ca 125 test was mildly elevated at 17.5 (normal 1.9-16.3 U/mL).

However, two weeks later, she returned to the Accident and Emergency department and was readmitted with similar complaints and examination findings. Vaginal examination also revealed a copious amount of malodorous yellow discharge. The cervix was also hyperaemic with contact bleeding. A high vaginal swab was taken at that point and she also had repeat urine and blood cultures. The patient was again admitted for stabilisation and treatment. She was again put on insulin and antibiotics ceftriaxone followed by ceftazidime, amoxicillin, and clavulanic acid. She was also seen by a gynaecologist and evaluated and investigated for staging laparotomy. Her liver function, renal function, intravenous pyelogram and barium enema were all normal. Her complete blood count was similar to that obtained before. Her blood cultures also grew methicillin-resistant *Staphylococcus epidermidis* (MRSE) in two bottles sensitive to ciprofloxacin, which was started. The vaginal swab results revealed *Trichomonas vaginalis*, no pus cells, 2+ Gram-positive cocci, 2+ Gram-positive bacilli and scant Gram-negative bacilli. However, the culture had no pathogens isolated. She was treated with antibiotics for 26 days and again improved with reduction in her spiking fever and improvements in her blood sugars. She was then scheduled for staging laparotomy and discharged home.

One month later, she was admitted for staging laparotomy and found to be ill-looking with pallor, fever and abdominal tenderness. Her Hb was 7.6 g/dL (normal range 11-15 g/dL), WBC 11 × 10^9^/L (normal range 4-11 × 10^9^/L). The anaemia was confirmed as anaemia of chronic disease with an elevated serum ferritin of 149.4 ng/mL (normal for premenopausal women 6-81 ng/mL). She was placed on insulin and antibiotics and transfused.

**Figure 2:**
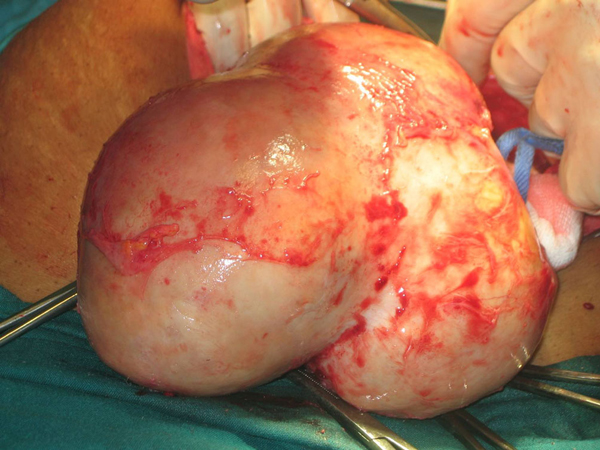
**A large pedunculated fibroid oozing pus**.

At staging laparotomy, she was found to have a large uterus with multiple fibroids. This uterus filled the abdomen and there was a pedunculated fibroid up under the right lobe of the liver. The uterus was noted to be very boggy as if fluid filled and was adherent to the anterior abdominal wall and the loops of bowel. By a combination of blunt and sharp dissection, the uterus was freed from the adhesions but in the process, it was inadvertently breached and copious amounts of purulent malodorous fluid (pus) oozed from it. This was suctioned (Figure 2) but not sent for culture and sensitivity. A subtotal hysterectomy and bilateral salpingo-oophorectomy were performed; the surgery was difficult and the patient gravely ill with heavy bleeding at surgery. The measured blood loss was 1.9 L.

The patient made a rapid recovery with an immediate decrease in her temperature and an improvement in her blood sugar control. Treatment with amoxicillin and clavulanic acid were continued post-operatively. She was discharged on the fifth postoperative day and is well at the time of writing, two years later.

**Figure 3:**
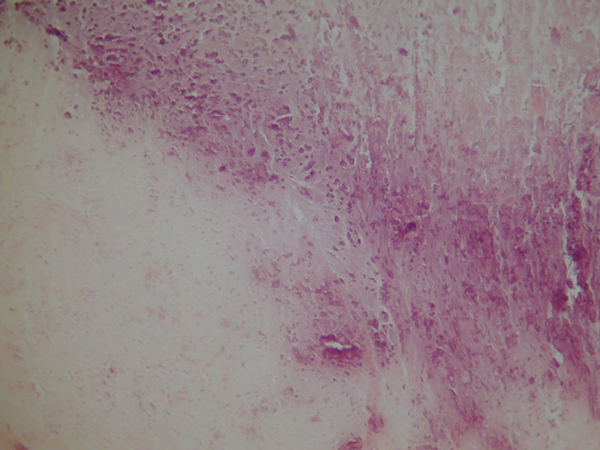
**A fibroid showing extensive calcification and infarction (haematoxylin and eosin, ×100)**.

Histologic examination of the fibroid showed extensive infarction and cystic degeneration (Figure 3). The endometrium was atrophic and both fallopian tubes were dilated consistent with hydrosalpinges. The ovaries were unremarkable.

## Discussion

Pyomyoma (suppurative leiomyoma of the uterus) is a rare disease, which is considered to be a serious complication of uterine leiomyoma. Since 1945, only 19 patients have been reported in the literature [3]. It is more usual in pregnant women or postmenopausal women who have vascular disease [2] or with cervical stenosis [4].

Pyomyoma is associated with a high fatality rate of about 21% [5]. Only surgical intervention is life saving [2]. The condition may be difficult to diagnose especially in those with a nonspecific clinical presentation without any history of leiomyoma. Delayed diagnosis may result in serious complications, whereas adequate surgery and broad-spectrum antibiotics may decrease serious morbidity and mortality [3]. The protracted course and myriad differential diagnoses in this patient support this point.

The condition usually results from infarction and infection of a leiomyoma [6]. Both conditions were clearly evident in this patient. The possible routes of infection for the development of pyomyoma have been described as contiguous spread from the endometrial cavity, direct extension from the adjacent bowel or adnexa, or haematogenous or lymphatic spread from infection elsewhere in the body [5,7].

The triad of: 1) bacteraemia or sepsis; 2) leiomyoma uteri; and 3) no other apparent source of infection should suggest the diagnosis of pyomyoma [7]. In this case, the patient had bacteraemia (MRSE) and no other obvious focus of infection as no other specimens grew pathogens. Staphylococcus is a common cause of abscess formation especially in an immunocompromised host. The presence of what appeared to be large fibroids clinically and on ultrasonography, completed the triad. Diagnosis was made more difficult by the fact that the fibroids were fluid filled on the later ultrasound scan as well as the CT scan (Figure 1). In one similar case, the patient was perimenopausal and had an intra-uterine device in situ. She was diagnosed as having infected malignant ovarian cancer with an elevated CA 125 level, and was initially treated with broad-spectrum antibiotics; she then underwent total abdominal hysterectomy and bilateral salpingo-oophorectomy. Pathological findings showed acute and chronic inflammation of the endometrium with abscess formation in an intramural leiomyoma [3]. In another case report almost identical to ours, a diabetic postmenopausal woman with a giant pyomyoma simulating an ovarian cancer was described, based on the CT findings of a multicystic mass arising from the pelvis [5].

In other cases, the causes were illicit drug use causing transient bacteraemia resulting in bacterial seeding of a uterine leiomyoma [8] and instrumentation at dilatation and curettage [9].

In this woman, the presence of chronic pelvic inflammatory disease and recent coital activity is suggestive of a spread of infection to the fibroid from the fallopian tubes. The fact that she had diabetes mellitus made the patient more susceptible to infection. The infection led to difficulty in controlling the diabetes; hence a vicious cycle was created. This was stopped by surgical intervention.

Patients with methicillin-resistant staphylococcus are also resistant to cephalosporins in many cases. These were the two types of antibiotics given to this patient. Patients with these infections are also known to have a poorer prognosis than patients who have other bacterial infections [10]. The bacteraemia in this patient was due to MRSE which is less potent than its better known cousin, methicillin-resistant *Staphylococcus aureus* (MRSA).

Pyomyoma should not be confused with a pyometra which is an accumulation of purulent material in the uterine cavity, another more common pathologic entity. With pyometra, the diagnosis is based on the classic symptoms of uterine enlargement, vaginal discharge and acute abdomen. This also requires rapid supportive therapy and surgical intervention [10].

## Conclusion

This is an excellent teaching case to remind readers of pyomyoma. The large fibroids, the pyrexia of unknown origin, the diabetic perimenopausal woman and the classic CT findings of fibroids with a cystic centre [4,11] all point to pyomyoma. We hope this case report will help some readers make the diagnosis earlier in their patients.

## Abbreviations

CA 125: cancer antigen 125 or Carbohydrate antigen 125; CT: computed tomography; Hb: haemoglobin; HIV: Human Immunodeficiency Virus; MRSA: methicillin-resistant *Staphylococcus aureus*; MRSE: methicillin-resistant *Staphylococcus epidermidis*; WBC: white blood cell count.

## Consent

Written informed consent was obtained from the patient for publication of this case report and any accompanying images. A copy of the written consent is available for review by the Editor-in-Chief of this journal.

## Competing interests

The authors declare that they have no competing interests.

## Authors' contributions

HF was the surgeon who managed the case and wrote the manuscript. RG was the assistant surgeon who managed the case. NW was the pathologist who looked at the specimen and helped to write this manuscript. GW was the haematologist who managed the patient on the ward and helped to write the manuscript. AN was the microbiologist who managed patient and helped to write the manuscript. DS was the radiologist who carried out scans and provided images of the CT scan.
